# Acid ceramidase gene therapy ameliorates pulmonary arterial hypertension with right heart dysfunction

**DOI:** 10.1186/s12931-023-02487-2

**Published:** 2023-08-11

**Authors:** Michael G. Katz, Yoav Hadas, Adam Vincek, Lina Freage-Kahn, Nataly Shtraizent, Jeko M. Madjarov, Peter Pastuszko, Efrat Eliyahu

**Affiliations:** 1 Department of Genetics and Genomic Sciences, Icahn School of Medicine at Mount Sinai, 1470 Madison Ave, P.O. Box 1030, New York, NY 10029-6574 USA; 2 Department of Pediatric Cardiac Surgery, Icahn School of Medicine at Mount Sinai, New York, NY USA; 3 Department of Psychiatry, Icahn School of Medicine at Mount Sinai, New York, NY USA; 4 Aveta.Life, Hoboken, NJ USA; 5 SeneX Therapeutics Inc., New York, NY USA; 6 Atrium Health Sanger Heart and Vascular Institute, Charlotte, NC USA; 7 Wake Forest School of Medicine, Winston-Salem, NC USA; 8 Icahn Genomics Institute, Icahn School of Medicine at Mount Sinai, New York, NY USA

**Keywords:** Pulmonary arterial hypertension, Gene therapy, Sphingolipid metabolism, Adeno-associated virus, And acid ceramidase

## Abstract

**Background:**

Up-regulation of ceramides in pulmonary hypertension (PH), contributing to perturbations in sphingolipid homeostasis and the transition of cells to a senescence state. We assessed the safety, feasibility, and efficiency of acid ceramidase gene transfer in a rodent PH model.

**Methods:**

A model of PH was established by the combination of left pneumonectomy and injection of Sugen toxin. Magnetic resonance imaging and right heart catheterization confirmed development of PH. Animals were subjected to intratracheal administration of synthetic adeno-associated viral vector (Anc80L65) carrying the acid ceramidase (Anc80L65.AC), an empty capsid vector, or saline. Therapeutic efficacy was evaluated 8 weeks after gene delivery.

**Results:**

Hemodynamic assessment 4 weeks after PH model the development demonstrated an increase in the mean pulmonary artery pressure to 30.4 ± 2.13 mmHg versus 10.4 ± 1.65 mmHg in sham (p < 0.001), which was consistent with the definition of PH. We documented a significant increase in pulmonary vascular resistance in the saline-treated (6.79 ± 0.85 mm Hg) and empty capsid (6.94 ± 0.47 mm Hg) groups, but not in animals receiving Anc80L65.AC (4.44 ± 0.71 mm Hg, p < 0.001). Morphometric analysis demonstrated an increase in medial wall thickness in control groups in comparison to those treated with acid ceramidase. After acid ceramidase gene delivery, a significant decrease of pro-inflammatory factors, interleukins, and senescence markers was observed.

**Conclusion:**

Gene delivery of acid ceramidase provided tropism to pulmonary tissue and ameliorated vascular remodeling with right ventricular dysfunction in pulmonary hypertension.

**Graphical Abstract:**

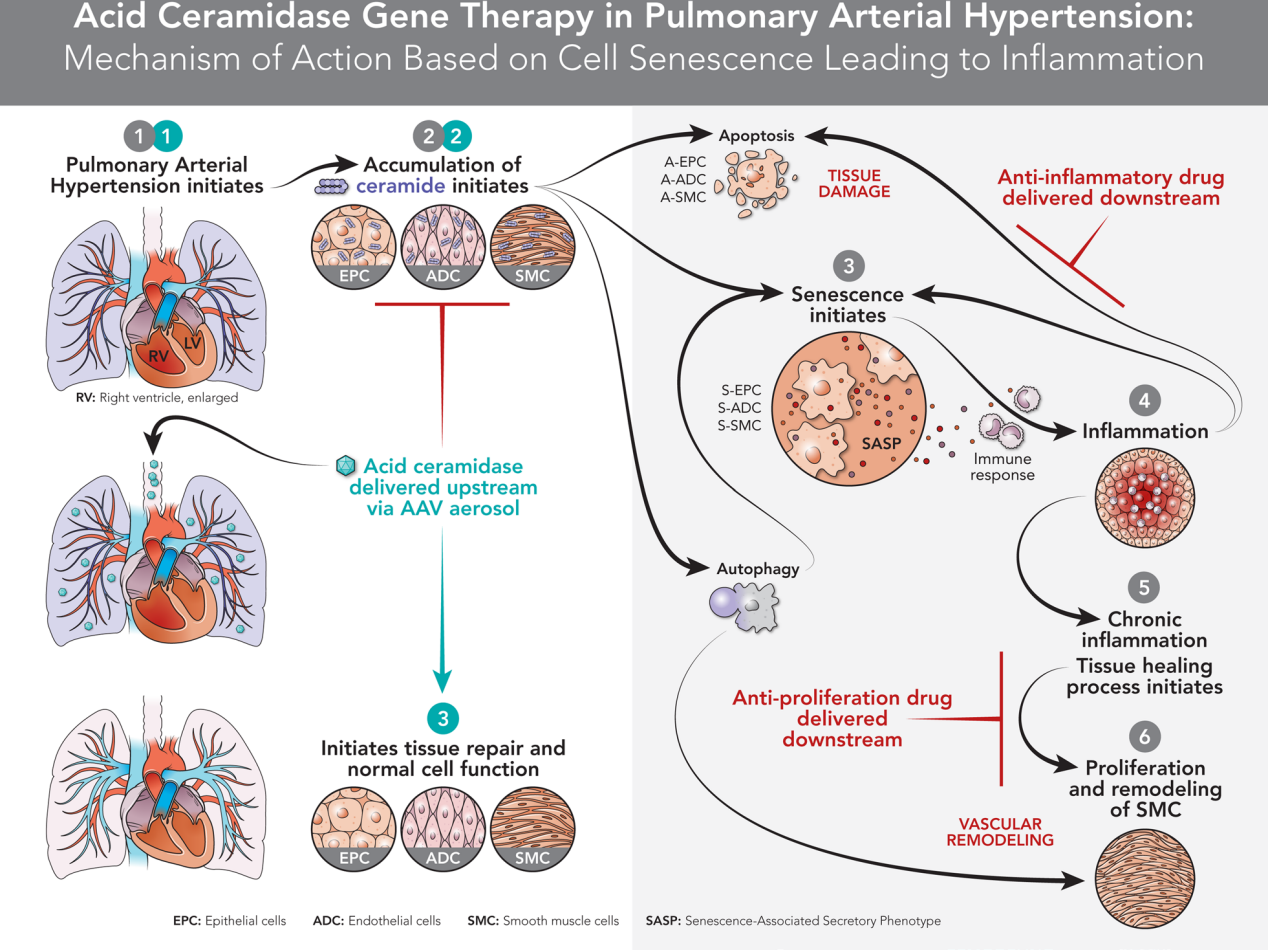

**Supplementary Information:**

The online version contains supplementary material available at 10.1186/s12931-023-02487-2.

## Background

Pulmonary hypertension (PH) is a progressive disorder of the pulmonary vasculature and is characterized by neointimal proliferation and smooth muscle cell hypertrophy, leading to vascular remodeling with elevation of pulmonary vascular resistance (PVR). A high PVR increase afterload that eventually causes intolerant right ventricular failure and premature death. In the United States more than 250,000 hospitalizations occur annually with pulmonary hypertension as a primary or secondary diagnosis [1]. Currently, the mortality rate from patients diagnosed with PH is 21% at 3 years, increasing to 47% among patients with comorbidities this suggests the importance of utilizing new therapies in the treatment [2]. The majorities of FDA-approved therapies currently available for PH are extremely expensive and attempt to overcome the disparity between vasoconstriction and vasodilation to restore endothelial cell function. None of these medications have an effect on the molecular dysfunction of the cells or can halt the progression of pulmonary vascular and right ventricular remodeling. The ultimate treatment remains lung transplantation, as there are currently no therapies to treat major complications of the PH such as right ventricular (RV) failure.

PH is a multifactorial disease involving several biological processes including cellular metabolism that is the most predominant factor controlling the cell faith. The female predisposition to PAH has given rise to the hypothesis that female sex hormones, primarily estrogens, may play a causative role in the development of the condition (female/male 4:1). Estrogen can also induce proliferation of human pulmonary artery smooth muscle cell and may therefore contribute to the pulmonary artery remodeling observed in PH [3]. The exploration of bioactive lipids, especially sphingolipids representing one of the primary used studies in molecular cell biology [4]. Sphingolipids mainly, ceramide is structural and functional components in many cell pathways. The central role of ceramide in oxidative stress, initiating apoptosis, senescence, cell growth, inflammation and proliferation led to an investigation of its role in different pulmonary diseases [5–7]. Studies that investigated ceramides in this context concentrated on proliferation and trans differentiation of alveolar epithelial cells [8]. Acid ceramidase (AC) is the main enzyme that hydrolyzes ceramide to generate free fatty acids and sphingosine. A derivative of sphingosine, sphingosine-1-phosphate (S1P), can counteract the apoptotic effect of ceramide, leading to the suggestion that AC can be “rheostats” that maintain a balance between cell growth and death [9]. AC can induce both: reduce the apoptotic/senescence effect by hydrolyzing ceramide and increase the S1P levels initiating the survival effect. Recently it was found that sphingosine kinase1 (SK1)/S1P axis is activated in pulmonary smooth muscle cells in patients with idiopathic PH [10]. In contrast it was demonstrated that reduction of SK1 activity could contribute to the development of PH [11], and pulmonary artery endothelial cells apoptosis and formation of occlusive pulmonary lesions in a rat model of PH is due to inhibition of SK1/S1P signaling [12]. An AC deficiency study in mice, resulted in a significant impairment in lung function, including low compliance and increased airway resistance [13]. It was reported that AC deficiency leads to chronic lung injury with inflammation, increased vascular permeability, and changes in surfactant activity [13]. Furthermore, research studies indicate that AC is required to normalize function of alveolar macrophages and contribute to capillary gas exchange [14]. Based on prior evidence that showed an increase in ceramide levels in the lungs of patients with idiopathic PH, it is also reasonable to assume that AC might be a significant molecular factor required for down-regulating ceramide levels in PH. To gain more insight into the role of AC in the pathogenesis and treatment of PH, we investigated whether the level of AC was decreased in Sugen/pneumonectomy rat’s model and examined the effect of AC gene overexpression on the occlusive pulmonary arteriopathy and cardio-pulmonary function.

The efficient delivery of AC is a vital issue for clinically relevant therapy. The advantage of recombinant AC delivery with inhalation was demonstrated in mice [15]. The use of viral vectors, which are naturally evolved vehicles, can effectively transfer genes into a host cell. We believe that gene therapy with AC gene encoding by viral vector is more efficient and has a high potential to treat chronic lung disorders. Previously we demonstrated that synthetic adeno-associated virus, Anc80L65 is an effective gene transfer tool for the cardio-vascular system [16]. To facilitate the translation of current observations on the role of AC in PH pathobiology this study will explore the hypothesis that administration of AC mediated by Anc80L65 can modulate PH phenotype and right ventricular remodeling.

## Methods

### Animal’s studies and in vivo design

**Fig. 1:**
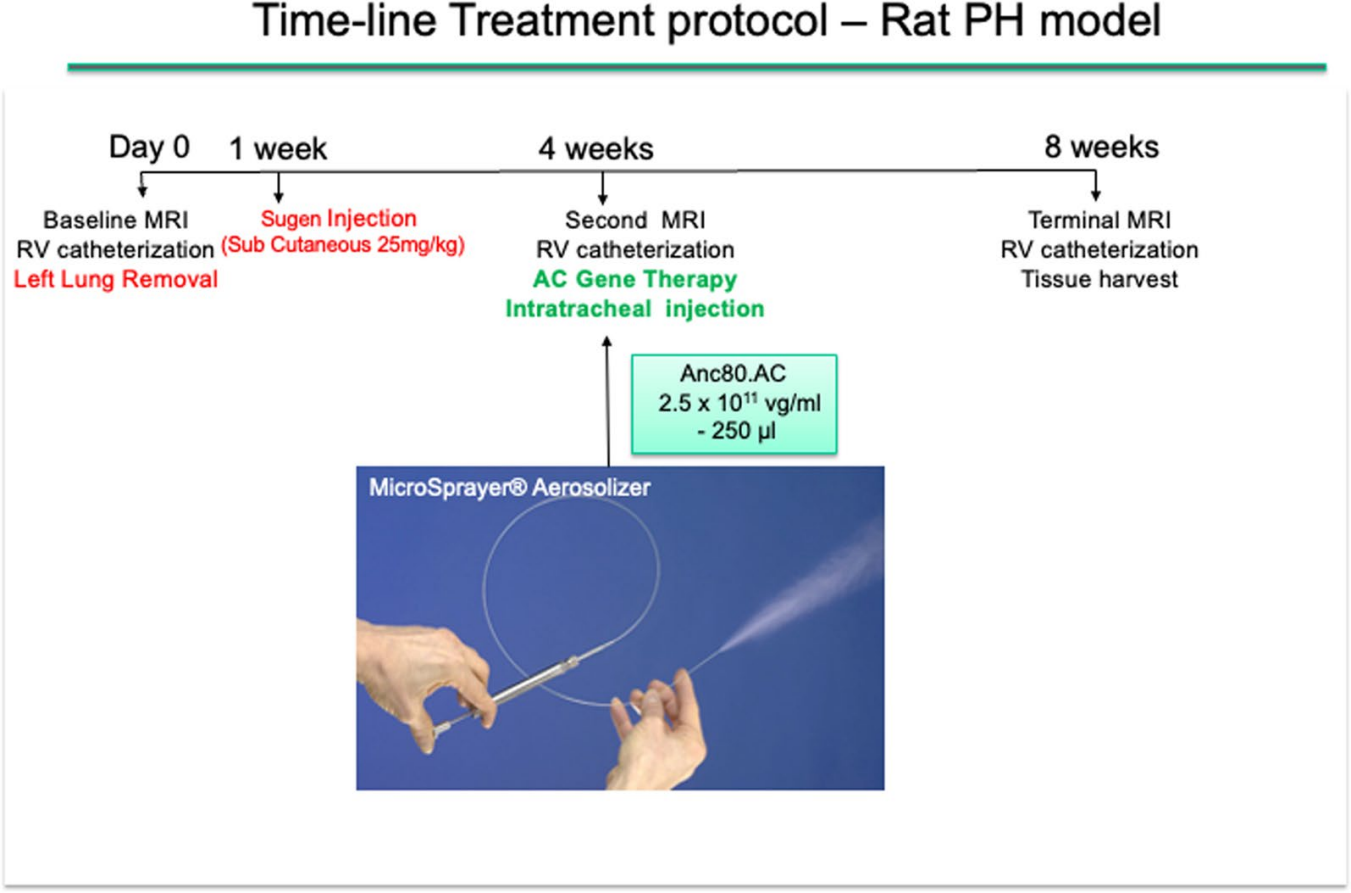
A flow chart of time-line protocol

A total of 56 Sprague–Dawley rats, 6–7 weeks, weighting 250–300 gr (Charles River Laboratories, Wilmington, MS) were used. Rats were randomly divided among five groups: sham (group 1); SU5416 (Sugen) + pneumonectomy (PNx), (PH, group 2); PAH and Saline (PH.Sal, group 3); PH and Anc80L65.Null (PH.Anc80.Null, group 4); PH and Anc80L65.AC (PH.Anc80.AC, group 5). To test the therapeutic potential of AC-based gene therapy for PH, we established a rodent’s model of PH by combination of left pneumonectomy with administration of SU5416 (Sugen) resulting in fast development of right heart failure and severe pulmonary vascular remodeling. A PH experimental model was adapted from Happe et al. [17] with our modifications [18] (Additional file 1: Video S1). In all rats baseline MRI and right ventricular (RV) catheterization with measurement pressure in pulmonary artery were performed. Then animals in groups 2–5 were subjected to the left lung removal. In-group 1 (sham) rats were subjected to thoracotomy only. One week later rats in-group 2–5 was subcutaneously injected with SU5416 (Sugen, 25 mg/kg; Sigma-Aldrich, St. Louis, MO) dissolved in carboxymethylcellulose. All animals were validated by MRI and right heart catheterization to confirm PH at 4 weeks time point. Then the rats were randomly assigned to receive either a single dose of Saline (250 μL), Anc80L65.Null, (2.5 × 10^11^ μg/ml), or Anc80L65 encoding human AC (2.5 × 10^11^ μg/ml) or nothing. The differences in numbers of animals in each group (8–10) are explained by technically challenging to make pulmonary artery catheterization and MRI in terminal procedures together. Treatments were performed with intratracheal-aerosolized delivery with amount of 250 µL using an IA-1C microsprayer (PennCentury, Wyndmoor, PA). A flow chart of time-line protocol is presented in Fig. 1.

### Surgical procedure (left pneumonectomy)

The animals anesthetized with isoflurane 3–4%, (1:1 oxygen/air mix) during 5 min, placed in dorsal recumbence. Then the animal is intubated (teflon tube 16 gauge) and ventilated throughout the procedure with isoflurane (2–2.5%) at 50–70 strokes per min and 2.5–3.5 ml tidal volume and transferred on a heating pad. Heart rate, oxygen saturation and temperature are monitored at all times during the surgery. The animal is then positioned in a left decubitus position and chest prepped in sterile fashion. A left postero-lateral thoracotomy incision over the third intercostal space is used. The superior and inferior pulmonary veins, pulmonary artery, and bronchus are isolated, stapled and tied. After that left lung is removed and hemostasis is achieved, closure of the ribs and intercostal muscles, and skin was beginning. After cessation of isoflurane, when the animal started spontaneous breathing efforts, the endotracheal tube was removed, and the rat was disconnected from ventilator. All data analysis was performed in a blinded manner. We have chosen to use rats in this proposal because extensive literature exists that rats are more susceptible to develop PH than other species, especially mice.

### Gene construct production

Anc80L65.Null and Anc80L65 encoding human AC viral stocks were prepared at the Gene Transfer Vector Core at Mass General Brigham Hospital (https://www.vdb-lab.org/vector-core/). Vector production, purification, characterization, and tittering are described in detail in other publications [19, 20].

### Magnetic resonance imaging (MRI) assessment of cardio-pulmonary hemodynamics, and heart dimensions

Cardiac magnetic resonance imaging (MRI) was performed at three time-points: baseline, 4 weeks, and 8 weeks post-pneumonectomy. For MRI, the rats were transferred on the instrumentation panel bed and loaded into the Bruker Small Animal 7T MRI unit (BRUKER AXS, Inc., Madison, WI). Respiration-gated MRI images was performed in the short and long axis. The images were acquired with a slice thickness of 1.5 mm, ensuring the entire biventricular length is covered. All DICOM images were loaded into the SEGMENT MRI software and analyzed by a certified blinded analyst. The short axis and long axis CINE series were analyzed for dimensions, volumes, ejection fraction, mass, regional function, and chamber indices via semi-automated contour segmentation.

### MRI analysis

Scans were coded by number and analyzed in batches by a radiologist who was blinded to the identity and hemodynamic results at the time of analysis. Stroke volume was determined from end-dyastolic volume (EDV) − end-systolic volume (ESV) of the LV. Ejection fraction (%) = (SV/EDV) × 100) was also determined. RV and LV masses were determined by manual planimetry at diastole. Ventricular mass index (VMI) was defined as the ratio between RV to LV mass, with the interventricular septum considered part of the LV. All ventricular volumes and mass measurements were indexed to body surface area.

### Pulmonary artery catheterization and hemodynamic studies

Rats were anesthetized, intubated, and mechanically ventilated. A 1.2 Fr transonic pressure catheter (Transonic Systems Inc., London, Ontario, Canada) was inserted into the pulmonary artery for direct measurements of pulmonary artery pressure, as previously described [21]. Hemodynamic data were recorded using an ADVantage PV Control Unit. Mean PA pressure (mPAP) was calculated by the formula: mPAP = (PA systolic pressure + 2 PA diastolic pressure/3). Pulmonary vascular resistance (PVR) was calculated as (mean PA pressure − left atrium pressure /cardiac output. Cardiac output was derived from MRI. For analyzing the tricuspid annular plane systolic excursion (TAPSE) we used MRI’s four-chamber views. The largest distance between the lateral tricuspid annulus and the right ventricular apex was defined at the end-systolic and end-diastolic stages. TAPSE was defined as the difference between those two measurements [22].

### Quantitative real-time quantitative polymerase chain reaction (qPCR)

QPCR was used to analyzed Anc80L65.Null and Anc80L65.AC vectors genome copy. Lung tissues were ground into powder in liquid nitrogen; the powder was then used for RNA extraction. Total RNA was isolated using Trizol Reagent (Thermo Fisher Scientific, Carlsbad, CA), and 500 ng were reverse transcribed according to the manufacturer’s instructions. Quantitative real time PCR amplification of cDNA was performed using the Perfecta SYBR Green FastMix (Quantabio). Total RNA was isolated using the RNeasy mini kit and reverse transcribed using Superscript III reverse transcriptase (Invitrogen). Real-time qPCR analyses were performed on a Mastercycler realplex 4 Sequence Detector (Eppendorf, Hauppauge, NY) using SYBR Green Quantitect TM SYBR Green PCR kit. Fold changes in gene expression were determined and presented relative to an internal control. The level of rat GAPDH DNA was used to calculate normalized transfection to obtain qPCR values. Absolute genomic copies of Anc80L65 and AC were quantified with the standard normal curve built from serial dilutions. Data were reported as viral genome copies per 1 μg of DNA.

### Western immunoblotting

Protein lysates (30 μg) were size-fractionated electrophoretically using SDS-PAGE, transferred onto a nitrocellulose membrane, blocked, and incubated with primary antibodies overnight at 4 °C. The membranes were incubated with anti-AC, anit-p21Cip1, anti-MMP3 and beta-Actin (Sigma, St. Louis, MO). Levels of proteins were detected by using horseradish peroxide-linked secondary antibodies (Cell Signaling, Beverly, MA) and the ECL System (Thermo Scientific, Rockford, IL) or the Odyssey CLx infrared imaging system (LI-COR, Lincoln, NE). Expression of AC, p21Cip1 and MMP3 was normalized relative to that of beta-actin.

### Sphingolipid composition

Mass spectrometry was used to determine the sphingolipid composition of frozen rat lungs crushed with a pestle that had been stored at − 80 °C. The powdered lung was transferred to cold Eppendorf tubes and snap frozen in liquid N_2_. Small volumes of Tris (20 mM, pH = 7.8) were added to the tubes, which were then subjected to sonication (10 min, 4 °C) for protein and lipid extraction. The tubes were centrifuged to remove the solid material, and the supernatants were transferred to clean tubes. Aliquots containing the equivalent of 1 mg total protein (BCA protein assay, Pierce™ A53226, 560 nM) were subjected to sphingolipid analysis in conical flasks (15 ml). Extraction solution (2 ml, ethyl acetate: isopropanol: water = 5:2.3:1) and internal standards (50 µL) were added to the flasks. Flasks were sonicated (3 s), vortexed (1 min) and centrifuged (5 min at 600 × *g*). Extracts were transferred to glass test tubes and a second extraction procedure was performed. The solvent was removed at 37 °C under a stream of N_2_ gas. Samples were resuspended in 150 µL of mobile phase B (methanol, formic acid (0.2%) and ammonium format (1 mM) for analysis.

### Inflammatory response and cytokine expression analysis

Concentrations of pro- and anti-inflammatory cytokines in sera were determined using a 23-plex assay (Bio-Plex Suspension Array System; Bio-Rad, Philadelphia, PA). Samples were diluted 1:4 in sample diluent and incubated for 30 min (room temperature, 300 rpm agitation) with capture antibody-coupled magnetic beads. After 3 washes in a Bio-Plex Pro wash station, samples were incubated for 30 min in the dark (room temperature, 300 rpm agitation) with bio-tinylated detection antibody. Each captured analyte was detected by the addition of streptavidin–phycoerythrin and quantified using a BioPlex array reader. Analyte concentrations were calculated with Bio-Plex Manager software.

### Immunofluorescence

Lung tissue was fixed with 4% paraformaldehyde. Tissue sections were incubated with rat primary polyclonal antibodies for AC (Santa Cruz Bio). Human antibodies conjugated with Alexa Fluor 488 or 594 were used as secondary reagents. Nuclear DAPI staining was performed by premixed DAPI with prolong gold anti fade mounting media. Fluorescence was visualized by a Zeiss LSM710 confocal microscope (Carl Zeiss GMBh, Jena, Germany) with 63 objectives. Image analysis was per-formed with the Zeiss ZEN 2010 image analysis software.

### Assessment of toxicity and blood gas analysis

Blood samples were collected from all rats and divided to fill 2 0.5 ml K3EDTA-containing tubes (Becton–Dickinson, Franklin Lakes, NJ). Samples were analyzed on an impedance hematology analyzer. The following variables were assessed: red blood cell count, hemoglobin concentration, mean corpuscular volume, total white blood cell count, percentage of neutrophils, lymphocytes, monocytes, and eosinophils. For blood gases, the samples were analyzed within 5 min as recommended by i-Stat1 technical bulletin using CG8 cartridges. All variables were determined using the VetScan i-Stat1 handheld analyzer (Abaxis, Union City, CA). The pH (hydrogen potential), pCO_2_, pO_2_, bases excess, HCO3, Na^+^, and K^+^ were carried out in all blood samples.

### Right and left ventricular weight measurement and determination of Fulton’s index

After the heart was excised, the right ventricular wall was dissected, and the remaining left ventricular wall and ventricular septum were weighed. Fulton’s Index was calculated as the ratio of right ventricular weight/(left ventricular + septum weight). The heart sections were then embedded in optimal cutting temperature (OCT) compound, and hematoxylin and eosin staining was performed on 8 μm–thick sections. Fibrosis was examined in frozen section (8 μm) that were fixed in 1% paraformaldehyde and stained with Masson’s trichrome stain. Sections were visualized.

### Heart and lung tissue histology and immunohistochemistry

Lungs were inflated with PBS, while heart and lung tissue were embedded in OCT, frozen, sectioned, and 8 μm sections were fixed with 1% paraformaldehyde. Sections were stained using hematoxylin and eosin, Masson’s trichrome and Verhoeff-van Gieson, and visualized using light microscopy for histological examination.

### Medial wall thickness and percentage of occluded vessels morphometry

Pulmonary arterial morphometry was performed as previously reported [23]. Transversally cut lung section (5 μm) were stained with Verhoeff-van Gieson for analysis of vascular dimensions. Only arteries with an external diameter < 200 μm and having a complete muscular coat were measured and assigned according to external diameter. The medial thickness was measured in distal PAs with the following formula: 100 × (external diameter − internal diameter)/external diameter. For each animal, medial thickness of at least 20 arteries was measured, and expressing thickness of the media layer as a percentage of total vessel diameters. The percentage of vessels occluded was calculated as a number of occluded vessels/50. Sections were visualized and quantified using ImageJ software.

### Transmission electron microscopy

Each field was scanned with a computer-generated microscale and analyzed blindly using randomly selected tissue sections from lungs with image analysis software. For lungs assessment, samples were analyzed by transmission electron microscopy (Hitachi 7000, Japan). Images were captured with a digital camera system. Samples were infiltrated with 1% aqueous osmium tetroxide in a BioWave Pro microwave, dehydrated in a graded series of ethanol in the BioWave Pro, and embedded in Spurr resin in a 70 °C conventional oven. Ultra-thin sections on copper grids were stained with 2% uranyl acetate and Sato’s lead citrate. Digital images were captured with a digital camera system from 4 pi analysis.

### Statistical analysis

Outcomes are presented as a mean standard deviation or median (interquartile range) based on normality. For cardiac hemodynamics and molecular biology assay data, 1-way analyses of variance were used for time-independent factors that satisfied the normality assumptions in all groups, and post hoc tests were used to assess differences between specific groups. Statistical comparisons for the box-and-whisker dot plots were made using the bees warm boxplot commands in R on the basis of 1-way analysis of variance comparing the median/50% percentile with multiple comparison tests. For statistical comparisons we used Tukey honest significant difference analysis using the Tukey HSD command in R with a confidence level of 0.99.

## Results

### Acid ceramidase gene therapy improved cardio-pulmonary hemodynamics and decreased right ventricular (RV) dysfunction

**Fig. 2:**
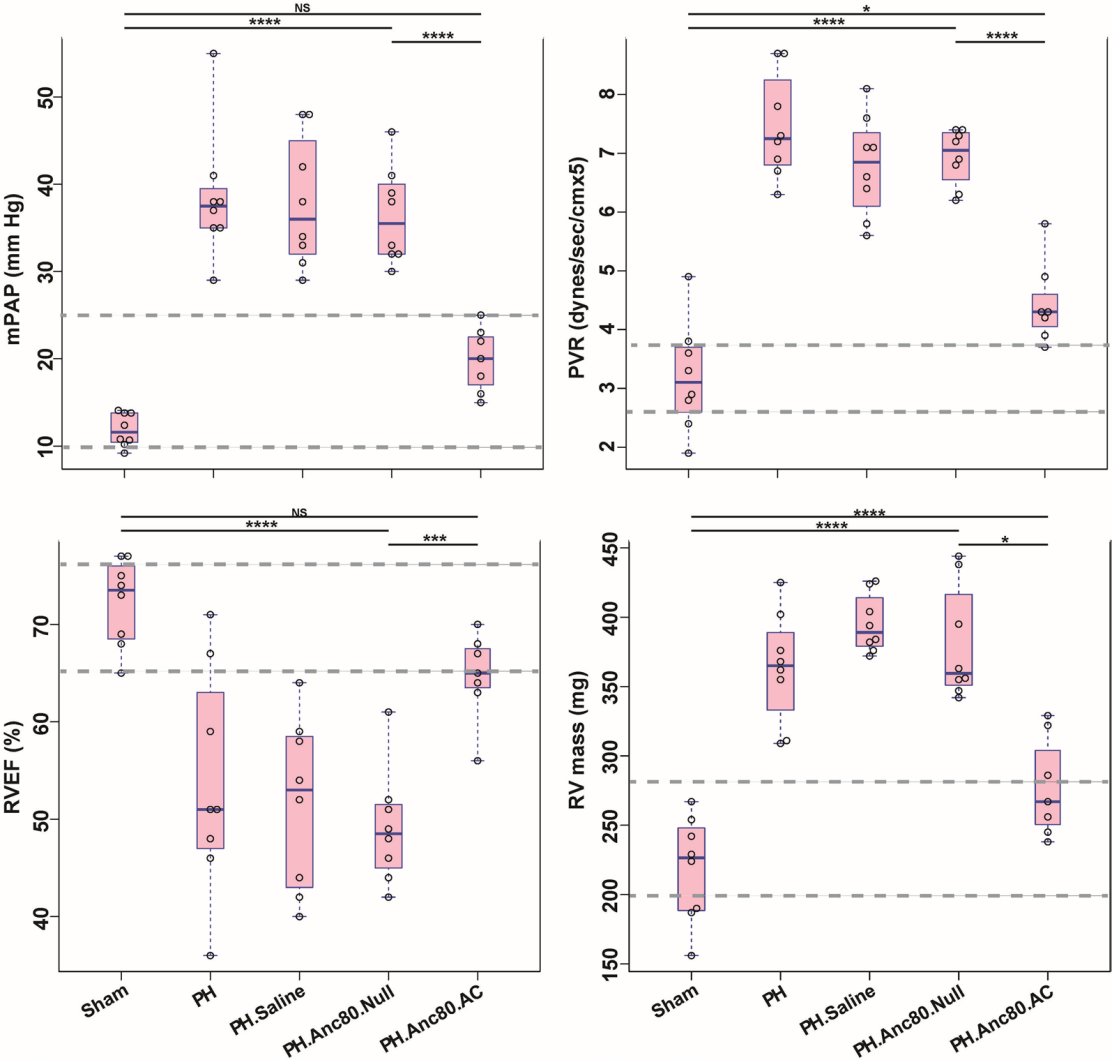
Evaluation of Anc80.AC effect on cardiopulmonary hemodynamic parameters and right ventricular function in rats 8 weeks post-PAH induction. Right ventricular (RV) hemodynamic parameters improved post gene therapy with Anc.80AC in PH model. **A** The mean pulmonary arterial pressure (mPAP, mm Hg). **B** Pulmonary vascular resistance index (PVR, dynes/sec/cmx5). **C** Right ventricular ejection fraction (RVEF, %). **D** Right ventricular mass (RV mass, mg). All continuous data were checked for normality and are presented as mean ± SD. Each group included 8 rats. Not significant p > 0.05; *p ≤ 0.05; **p ≤ 0.01; ***p ≤ 0.001; ****p ≤ 0.0001

Right heart catheterization 4 weeks after PH development in rats demonstrated increase in the mean PA pressure to 30.4 ± 2.13 mmHg from 10.4 ± 1.65 mmHg, which was consistent with definition of PAH in humans and in our previous rodent’s study [24, 25]. Also, at this time point, we documented a significant increase in PVR (6.13 ± 0.46 mm Hg, as compared with sham subjects displaying 2.78 ± 0.044 mm Hg, p < 0.0001. There were no significant differences with respect to heart weight, mean RV pressure, or heart rate in all groups at baseline (Additional file 2: Table S1). RV function was globally preserved, and rat’s behaviors were normal. In contrast, at 8 weeks PH rats showed signs of vital distress with RV dysfunction, accompanied by RV dilatation, decreased RV ejection fraction, and significant RV hypertrophy with an elevation of RV weight. Moreover, the number of clinical signs including noise while breathing, exercise-induced shortness of breath, elevation of white blood cells counts, and significant decrease in arterial PaO_2_ were present (Additional file 2: Table S2). Following endotracheal delivery of the gene construct at 8 weeks a final assessment of cardiopulmonary hemodynamics, vascular remodeling, and RV structure and function was performed. Analysis of heart function by MRI and RV catheterization in saline treated and Anc80.Null groups demonstrated that PH progression was associated with RV dysfunction (Fig. 2).

**Fig. 3:**
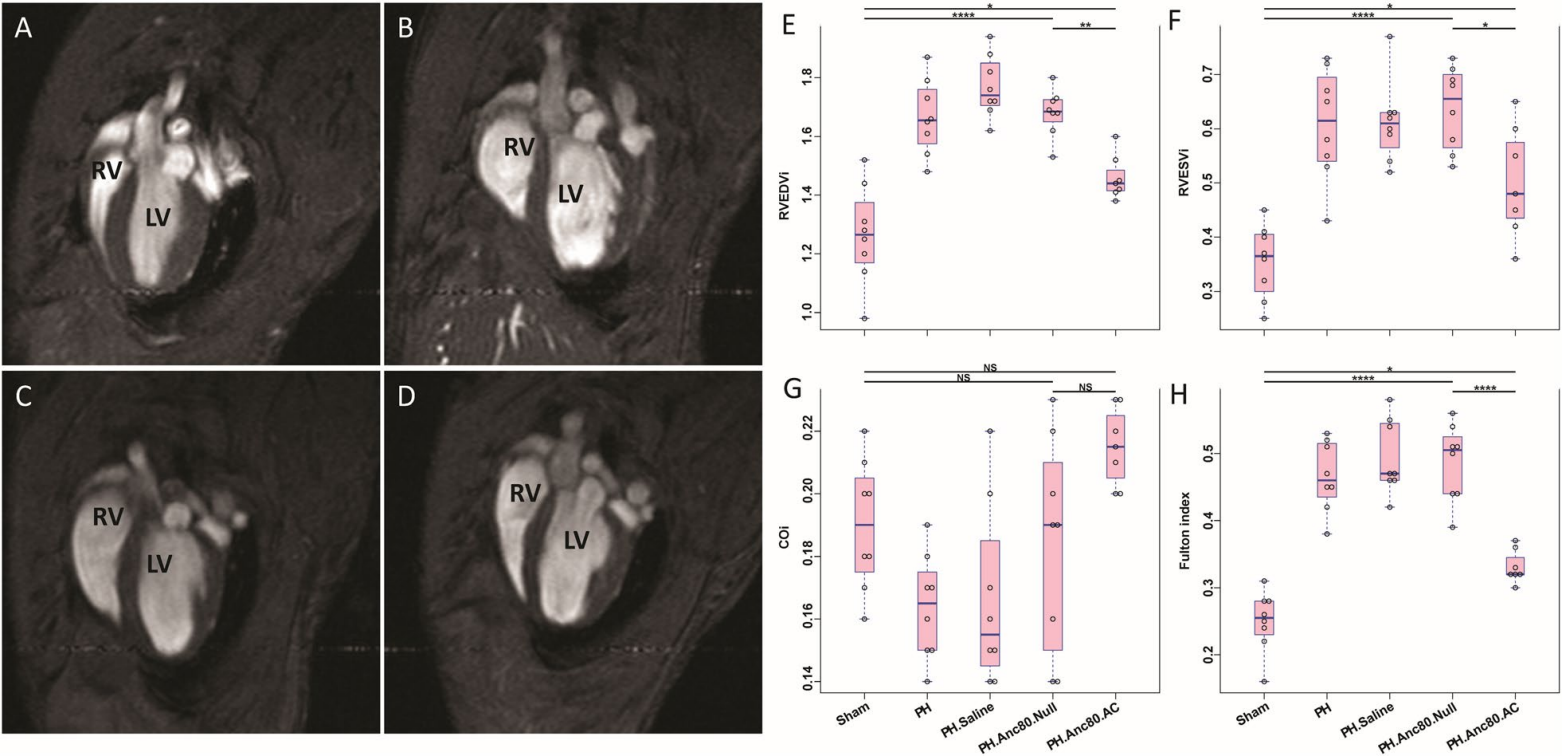
Evaluation of Anc80.AC effect on heart function and dimensions using cardiac magnetic resonance imaging (MRI) in rats 8 weeks post- PH development**.** MRI-based hemodynamic indexes have a tendency to return to normal values after AC gene therapy. **A** Representative MRI image of long-axis view of sham rat with normal cardiac function. **B** Representative MRI image of long-axis view of PH established rat with progressive RV dilatation and increased RV end-diastolic and RV end-systolic index. **C** Representative MRI image of long-axis view of PH.Anc80.Null treated rat with progressive RV dilatation and increased RV end-diastolic and RV end-systolic index. **D** Representative MRI image of long-axis view of PH.Anc80.AC treated rat with reduction of RV dilatation and decreased RV end-diastolic and RV end-systolic index comparing to PH induced untreated rat

Significant decrease of right systolic contractile function, as evidenced by impaired RV ejection fraction, and marked RV systolic and diastolic enlargement were found by MRI (Fig. 3).

**Fig. 4:**
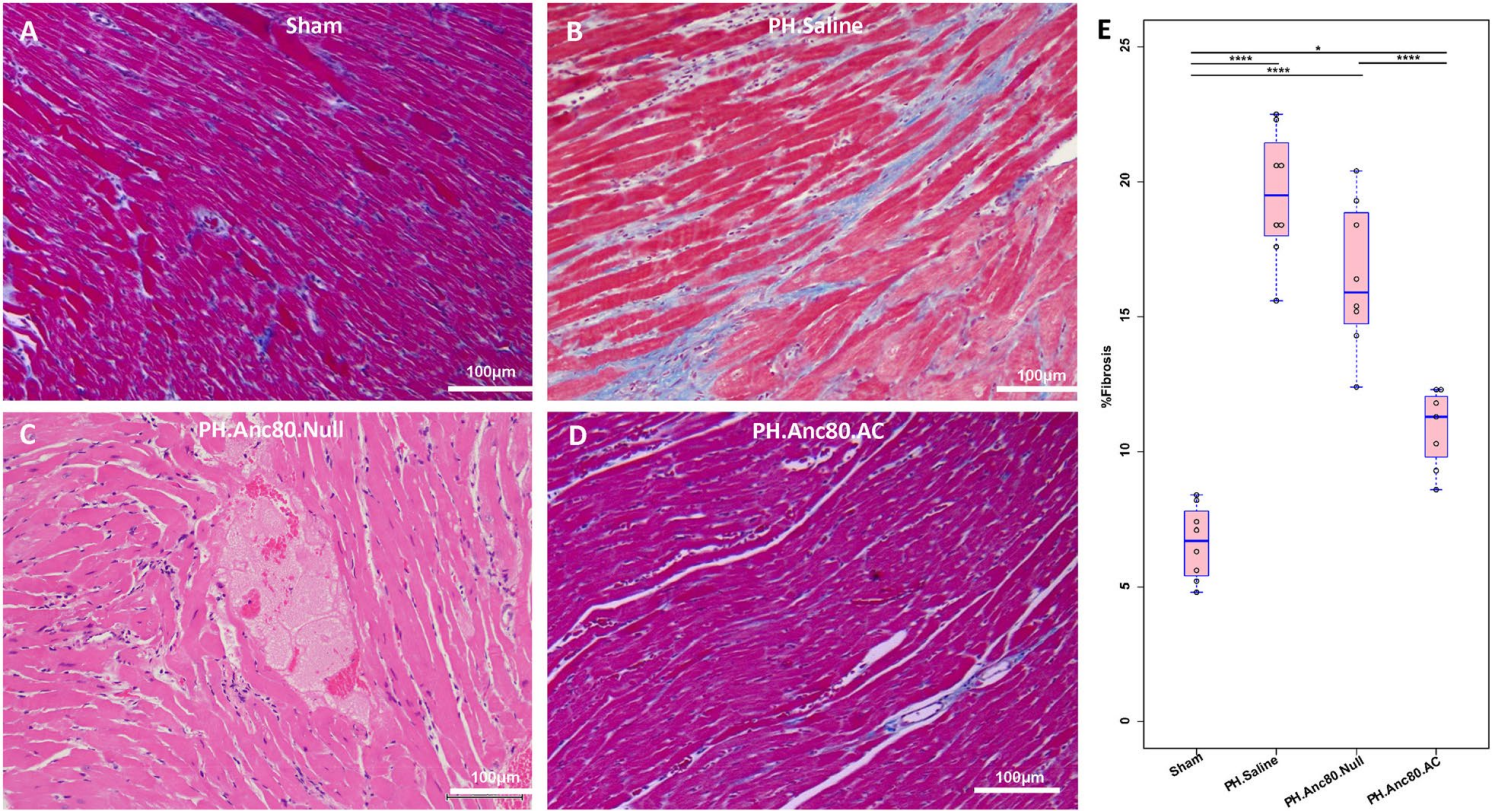
Right ventricular hypertrophy is ameliorated after Anc.80AC gene therapy in rats 8 weeks post-PH development. Representative hematoxylin–eosin staining is shown for: **A** Sham animals with normal RV structural organization. **B** PH.Anc80.Null. There is focally extensive replacement of myocardium by fibrous tissue with multifocal aggregates of lymphocytes and plasma cells. Also, there is degeneration of the myocytes adjacent to the fibrotic area characterized by swollen sarcoplasm, hypereosinophilia. **C** PH. Saline. Myocytes consistently had enlarged, hypertrophic and hyperchromatic nuclei, and myofibrillar disarray included cellular interplaying in various direction. Additionally, there is severe degeneration of myocytes with loss of cross striations. **D** PH.Anc80.AC. There is minimal to mild myxomatous change characterized by deposition of scant to mild amounts of amphophilic material and degeneration of few myocytes immediately adjacent. Rare lymphocytes are present in affected areas. Mild RV hypertrophic changes in this group of animals were seen. Bar scale: 100 μm and 200 μm

Corresponding to the observed changes, PVR was increased significantly in the saline treated and Anc80.Null group but not in animals receiving Anc80.AC, suggesting that AC treatment had a beneficial effect on vascular remodeling. Similarly, relative RV mass index was significantly elevated at 8 weeks compared to AC group and sham. In contrast, in the treatment group, AC improved RV systolic and diastolic function as well as ejection fraction. Histologic measurements confirmed the right ventricular hypertrophy assessed by light microscopy only in PH, saline treated and Anc80.Null groups (Fig. 4).

Overall, 6 rats were lost after developing right heart failure in groups 2–4 during follow-up period at 5–8 weeks. At necropsy, there was no lung infiltrates or infection; however, all 6 animals had evidence of severe right heart failure, including hepatomegaly and ascites, implicating this as the primary cause of death. No animal deaths were present in groups 1 and 5. Of the animals within the Anc80.AC group that completed the study, no signs of pulmonary infection or congestion were identified at necropsy. In all cases, aerosol endotracheal delivery of either Anc80.AC was well tolerated and did not result in respiratory or hemodynamic instability.

### Organ bio-distribution of Anc80.AC gene construct and AC expression

**Fig. 5:**
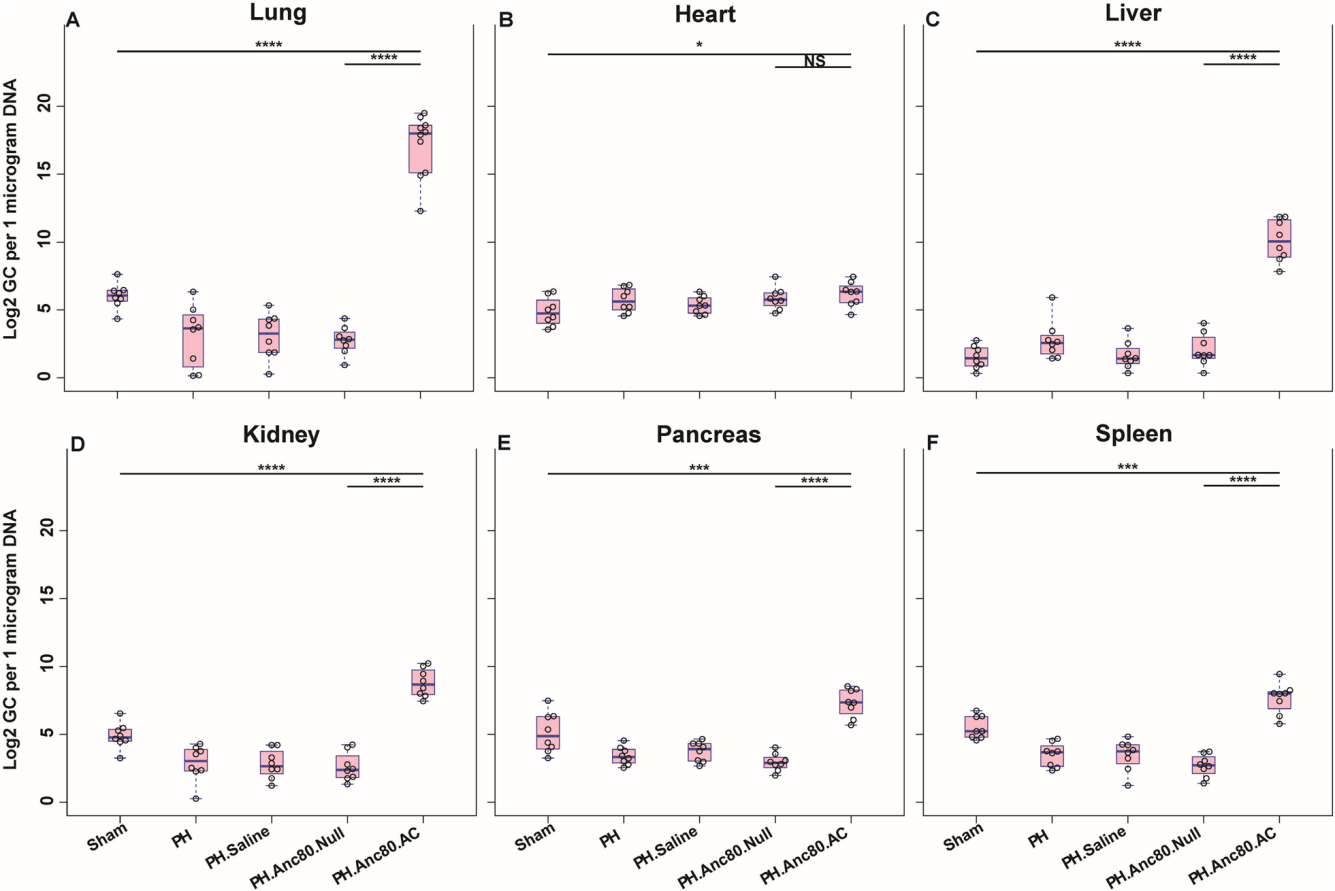
Organ’s viral cDNA Bio distribution of Anc80.AC in rats 8 weeks post-PH development**.** **A** Anc80.AC bio distribution in lungs. **B** Anc80.AC bio distribution in heart. **C** Anc80.AC bio distribution in liver. **D** Anc80.AC bio distribution in kidney. **E** Anc80.AC bio distribution in pancreas. **F** Anc80.AC bio distribution in spleen. All continuous data were checked for normality and are presented as mean ± SD. Each group included 8 rats. Not significant p > 0.05; *p ≤ 0.05; **p ≤ 0.01; ***p ≤ 0.001; ****p ≤ 0.0001

**Fig. 6:**
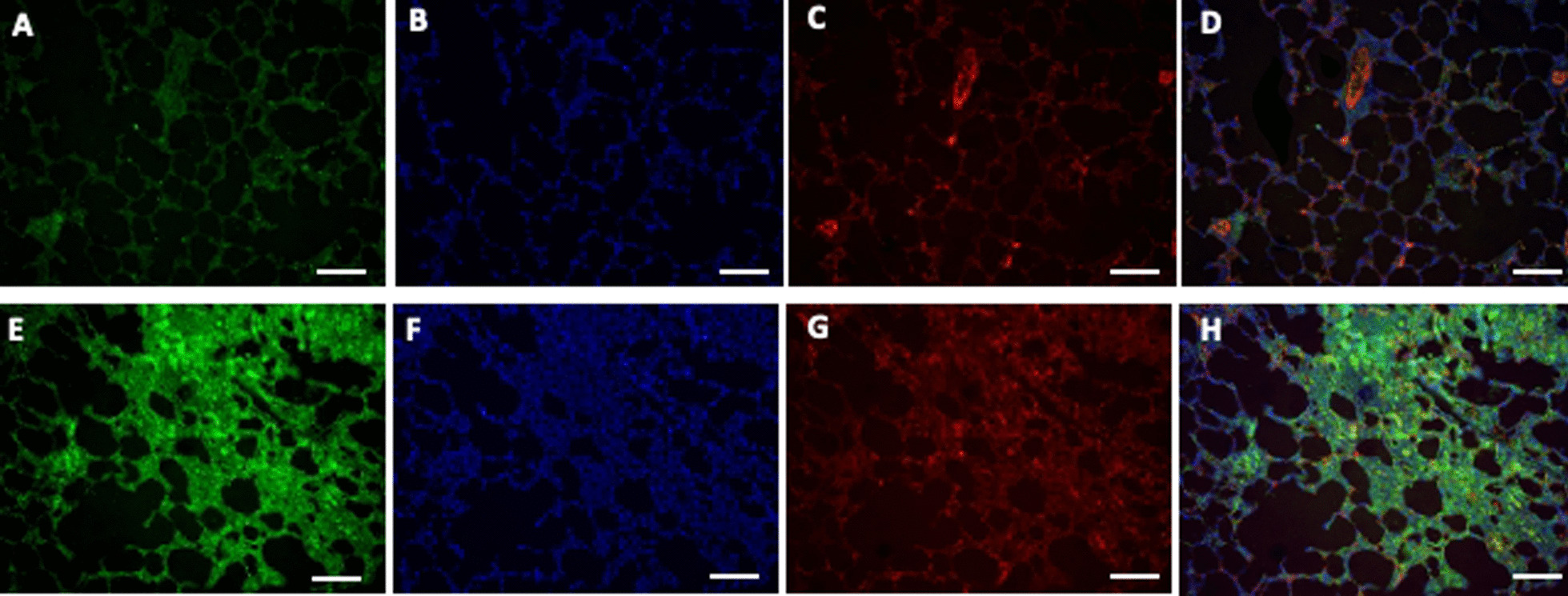
Immunofluorescence staining of AC protein and smooth muscle actin (SMA) in rat lungs 8 weeks post-PH development in control group (PH, untreated) and PH.Anc.80.AC group. Representative images showing AC protein (bright green dots) and nuclei were visualized by DAPI staining (blue dots). **A**, **E** AC protein expression. **B**, **F** DNA DAPI staining. **C**, **G** SMA staining. **D**, **H** merge image of AC and DNA labeling. **A–D** PH. An80.Null. **D–G** PH.Anc80.AC. Scale bar: 50 μm

Intratracheal gene delivery of Anc80.AC after PH establishment resulted in robust transfection as assessed by qPCR (Fig. 5), AC expression by confocal microscopy (Fig. 6) and western blot analysis (Additional file 3: Figure S1) that demonstrated significant increased expression of genome copies in the lungs compared to other organs. Quantification analysis indicated a significant difference in lung’s AC expression level between treatment and control groups (AC group, 17.14 ± 2.30 log^2^ gc per 1 μg of DNA versus control group Anc80Null, 2.74 ± 1.04 log^2^ gc per 1 μg of DNA, p < 0.0001). There was no significant difference in lungs expression between the control groups. Interestingly, the development of PH decreased expression of AC in the lungs compared to the sham group. AC expression in the heart was higher compared to sham, yet we did not find significant differences between treatment and control groups.

### Sphingolipid levels changes in PAH rat model and post AC treatment

**Fig. 7:**
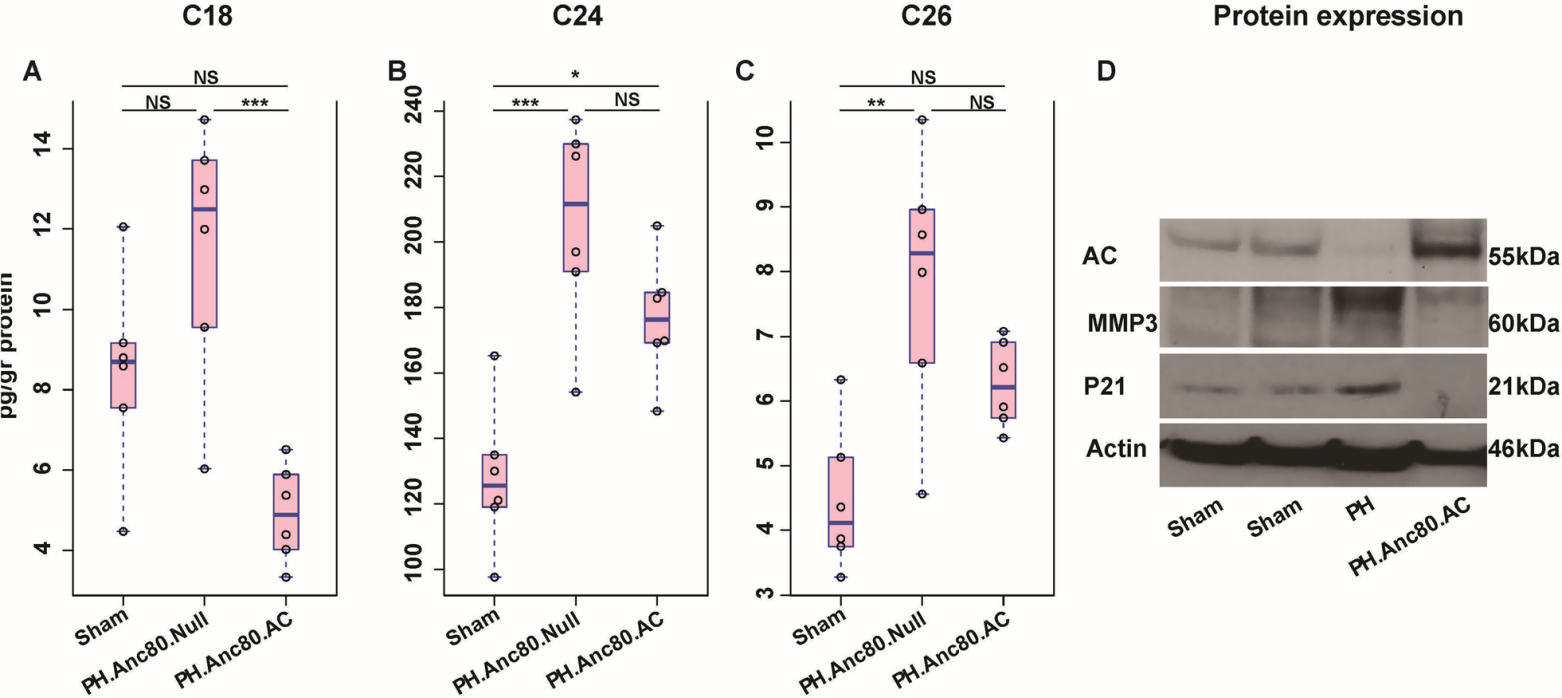
Assessment of main ceramides after Anc.80AC gene therapy in rats 8 weeks post-PH development. **A**–**C** Main ceramides level changes in Sham, PH.Anc80.Null and PH.Anc80.AC groups (pg/gr protein). AC gene therapy decreased level of main ceramide types. All continuous data were checked for normality and are presented as mean ± SD. Each group included 8 rats. Not significant p > 0.05; *p ≤ 0.05; **p ≤ 0.01; ***p ≤ 0.001; ****p ≤ 0.0001. **D** Representative western blot imaging of main senescence markers in lungs after Anc.80AC gene therapy in rats 8 weeks post-PAH creation. Senescence markers expression decreased after AC gene therapy. *P21* cyclin-dependent kinase inhibitor 1, *MMP-3* metalloproteinase-3, *kDa* kilodaltons, low molecular weight proteins

As was identified previously, mammalian ceramides contain a wide variety of fatty acids, ranging in length from C14 to C32 types [26]. We determined the main ceramide types levels in the lung at 8 weeks post-PH development. We found that the levels of main classes such as C18 (11.49 ± 3.21 pmol/ml), C24 (205.88 ± 31.50 pmol/ml) and C26 (7.83 ± 2.02 pmol/ml), were significantly higher in PAH compared to sham (p < 0.01) (Fig. 7). AC gene overexpression was able to significantly decrease this level in the treatment group (p < 0.001).

### Assessment of wall medial thickness, and percentage of occluded pulmonary vessels

**Fig. 8:**
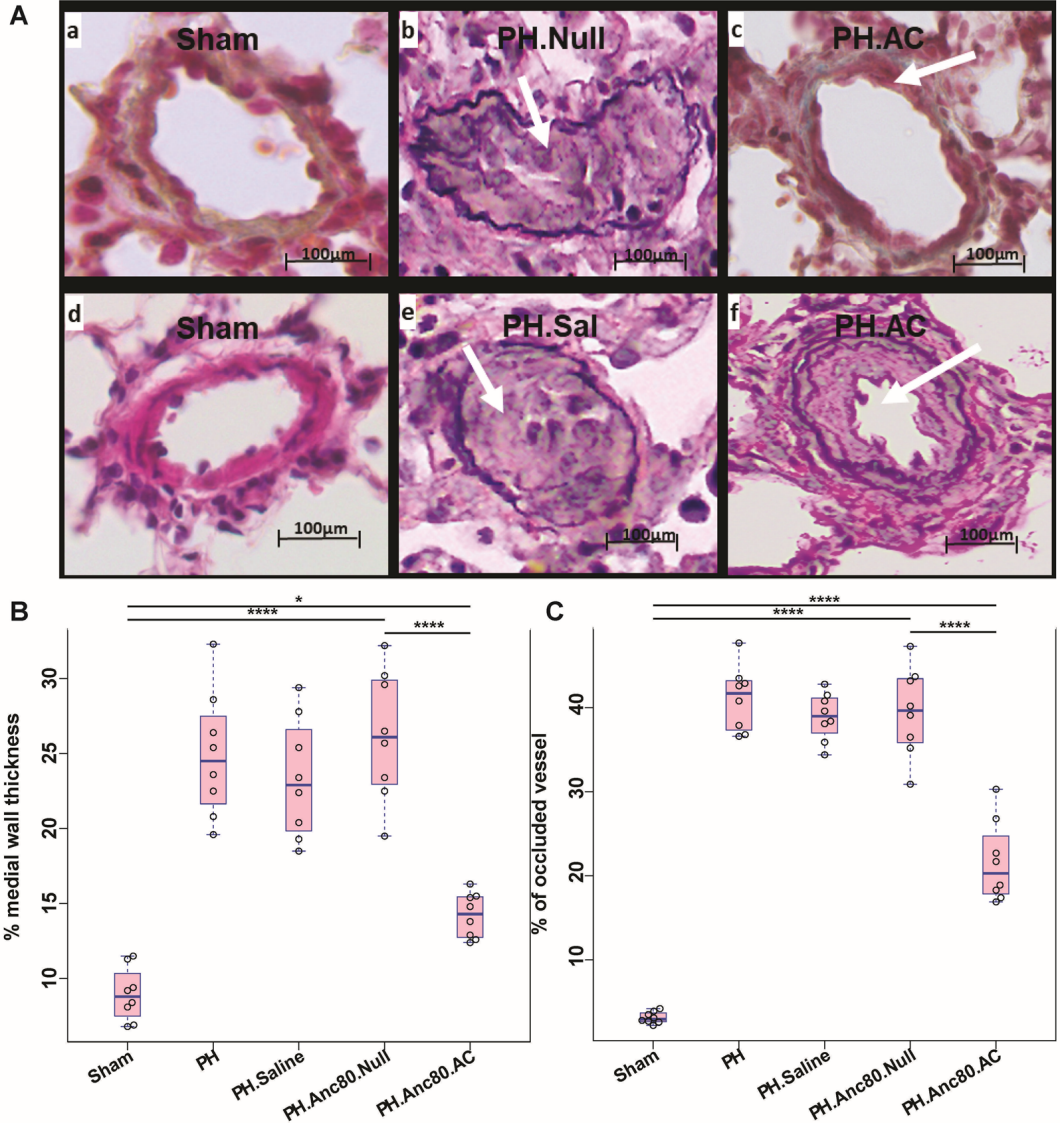
Histo-pathology results of the lungs in PH established untreated and PH established Anc.80AC treated animals. **Aa**–**Af** Representative hematoxylin–eosin and Verhoeff-van Gieson staining. **Aa**, **Ab** Sham animals; normal pulmonary arteries. **Ac**, **Ad** PH.Anc80.Null. Concentric laminar neointimal lesions. A cross-sectional view of completely occluded small pulmonary arteries by a complex lesions (grade 3–4). **Ae**, **Af** PH.Anc.80.AC. Mild neointimal reaction and proliferation, recanalization of small pulmonary artery (grade 1–2). **B**, **C** Percentage of occluded small pulmonary vessels, and medial wall thickness post Anc.80AC gene therapy in rats 8 weeks post-PH development. Scale bar: 100 μm. All continuous data were checked for normality and are presented as mean ± SD. Each group included 8 rats. Not significant p > 0.05; *p ≤ 0.05; **p ≤ 0.01; ***p ≤ 0.001; ****p ≤ 0.0001

Endotracheal gene transfer of AC partially reversed and limited pulmonary vascular remodeling in comparison with control groups. Morphometric analysis of distal pulmonary arteries demonstrated a significant increase in medial thickness in PH rats with no treatment in comparison with sham, and AC animals (Fig. 8). There was a statistically significant difference between the percentage of remodeled vessels between PH and AC groups. Immuno-histochemical analysis of small pulmonary arteries demonstrated vascular thickening and remodeling at 8 weeks. We found a statistically significant difference of the percentage of occluded vessels and medial wall thickness between Anc80.Null, and Anc80.AC groups (39.5 ± 5.26% versus 21.6 ± 4.81%; p < 0.001) and (26.2 ± 4.31% versus 14.21 ± 1.49%; p < 0.001 respectively).

### Lung changes as determined by electron microscopy

**Fig. 9:**
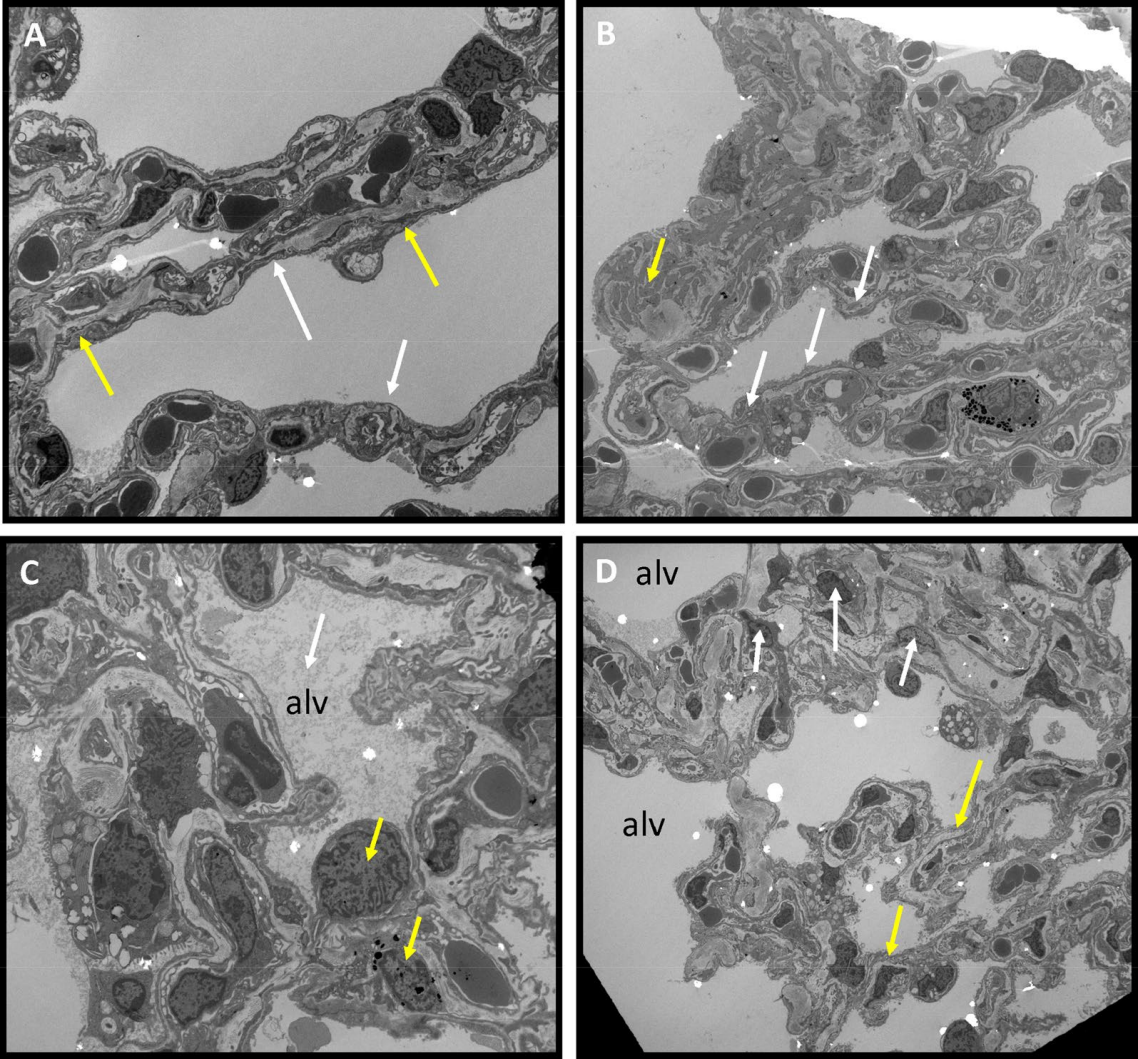
Transmission electron microscopy of PH established in control groups and PH.Anc.80AC treated rats 8 weeks post-PH development). **A** Sham. Normal, flat endothelial cells in the alveolo-capillary membrane separated from smooth muscle cells (white arrows) by thick basement membrane (yellow arrows). **B** PH.Anc80.Null. Intimal lesion displaying invagination of endothelial cells in the intima (white arrows). Swollen endothelium and thickened interstitium. Many collagens elastic fibers (yellow arrow). **C** PH.Saline Alveolar edema with fibrin in alveoli’s (white arrow). Presenting many inflammatory cells in interstitium. Degeneration of red and white blood cells with damaged the basement membrane (yellow arrow). **D** PH.Anc80.AC Endothelial layer denser and preserved (yellow arrow). Decreased number of inflammatory cells in the interstitium (white arrow), no alveolar edema. Bar scale: 10 μm, magnification: 2.500

Anc80.AC gene transfer protected the alveolar-capillary endothelial layer from significant injury. Electron microscopy micrographs showed that the sham group had a regular arrangement of endothelial cells, smooth edge of nucleus, and normal organelles distribution. Rats with PH exhibited the intimal invagination of endothelial cells with swelling and herniation into vascular lumen and irregular morphology of the nucleus, accompanied by a large amount of disorganized, proliferated collagen fibers and plenty of degenerated inflammatory cells. The cells showed irregularity and contained phagosomal vacuoles of varying sizes. Fibrin and fluid were seen in the alveolar lumen. In many regions, blood/gas barrier was thicker than normal due to an increase in thickness of capillary endothelial cells and its basement membrane. PH AC-treated rats showed a significant improvement in their endothelial layer, a practically normal nucleus shape, and decreased numbers of inflammatory cells (Fig. 9).

### Inhibition of senescence program initiated in PH and suppression of inflammatory cytokines activity by AC gene therapy

It was previously demonstrated that progression of PH is associated with cells senescence in pulmonary vasculature through 3 mechanisms: (i) a switch from a proliferative state into growth arrest; (ii) a shift from pro-apoptosis to apoptosis, and (iii) an increase in pro-inflammatory cytokines [27]. We confirmed senescence by demonstrating elevated expression in lung tissue using widely used senescence markers such as cell cycle inhibitor p21^Cip1^ and MM3-characterized cellular apoptosis. In the AC treatment group, we found decreased senescence markers based on western blot analysis (Fig. 7D). Previous studies have proven that cellular senescence is associated with chronic inflammation and that senescent cells actively secrete inflammatory mediators such as cytokines, which are involved in pulmonary vascular remodeling in PH [28, 29].

**Fig. 10:**
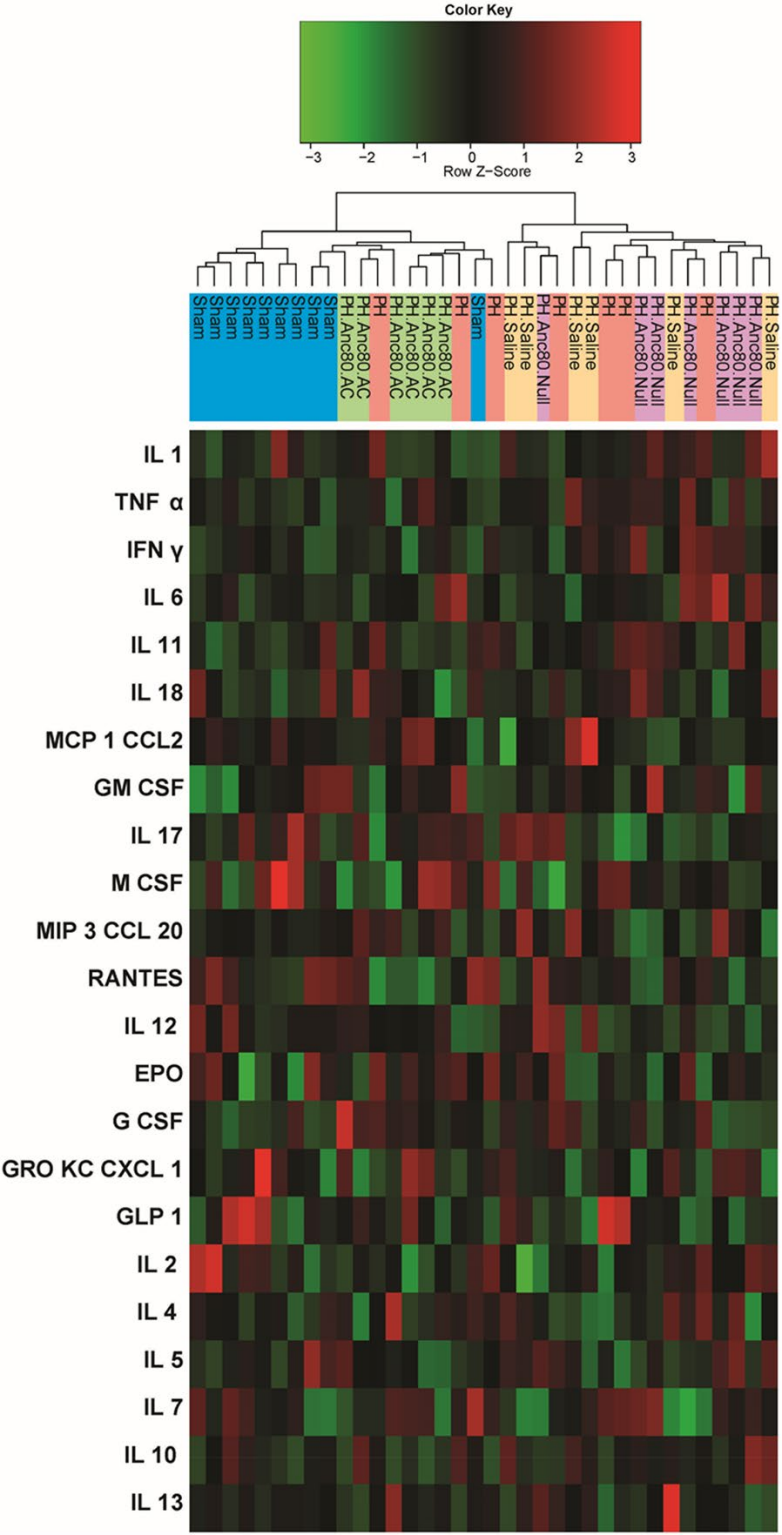
Changes in pro-inflammatory and anti-inflammatory cytokines expression at 8 weeks post-PH development. Among 23 cytokines investigated, a significant decrease of TNFα, INFγ, and interleukins 1, 6, 10, 12 were observed in Anc80.AC treated animals compared to untreated groups. The dendrogram shows the clustering of cytokines. The vertical line is name of cytokine. The horizontal line is group of the animals. The colors represent expression of IL, interleukin; EPO, erythropoietin; M CSF, macrophage colony-stimulating factor; MCP1 CCL2, monocyte chemoattractant protein; TNFα, tumor necrosis factor; G CSF, granulocyte colony-stimulating factor; IFNγ, interferon gamma; MIP3 CCL20, macrophage inflammatory protein; GRO KC CXCL 1, chemokine ligand; GLP 1, glucagon-like peptide; GM CSF, granulocyte–macrophage colony-stimulating factor

To observe possible changes in cytokine profiles in different groups, we performed cluster analysis. Based on the results of ensemble clustering the main cytokines such as TNFα, INFγ, and interleukins 1, 6, 10, 12 were significantly decreased in PH AC treated animals compared to non-treated groups (Additional file 4: Table S3). A heatmap and dendrogram for cytokines in serum is depicted in Fig. 10 and demonstrated the median of each cytokine and their differential expression pattern between the study groups. A comparison of cluster cytokine levels in Anc80AC and sham group showed very close cytokines profile at 8 weeks time-point. The level of pro-inflammatory cytokines was significantly higher in PH.Anc80.Null and PH. Saline groups (Additional file 4: Table S3).

## Discussion

Currently, PH is categorized into five distinct groups on the basis of pathophysiological and therapeutic commonalities [30]. Using both Sugen toxin and pneumonectomy we were able to combine the first PH classification group (drug and toxin induced) and third PH group (lung disease and/or hypoxia). We believe this model has many advantages over others and is much more similar to the human disease state due to following: (i) the model includes disturbed pulmonary blood flow as a trigger, causing plexiform lesions [31, 32], (ii) Sugen is lung-specific toxic kinase inhibitor that causes much less collateral damage than other toxic agent such as monocrotaline, and (iii) the model showed no reversibility of PH and RV hypertrophy, as in the most common models of Sugen/hypoxia.

Existing therapies cannot treat PH and RV dysfunction and can only improve the patient symptoms without stopping the disease progression [33]. Eventually, heart/lung transplantation will be required for patients who continue to progress. Although the histomorphology of PH is well described, the pathogenesis of the progressive senescence process and the angioproliferative lesions remain unknown. The most important reason for morbidity and mortality in PAH is the function of the right portion of the heart. RV failure is a common consequence of PH and is currently considered the strongest indicator of prognosis. It is well accepted that the spectrum of RV remodeling that occurs in PH ranges from adaptive remodeling, characterized by RV hypertrophy with overall preserved ventricular function, to maladaptive remodeling associated with RV hypertrophy, diastolic, and systolic dysfunction [34].

Increased understanding of the genetic basis for the development of PH leads to the consideration of adding gene-based approaches to current therapy. In the past years, several groups have explored gene therapy as a strategy to improve PH in different models. Targets of interest that have been studied include several of genes and viral vectors [35]. It should be noted that in recent years that AAV vector has gained popularity in translational medicine [36, 37]. In this study, we used a new synthetic AAV vector that does not induce toxic and inflammatory effects and provides efficient and early-onset expression [16]. Recently pulmonary specific drug transfers via inhalation devices have become an attractive therapeutic approach. Airway delivery has much lower endonuclease activities, which can destroy DNA and RNA molecules, as compared to intravascular route. Additionally, direct gene application can minimize systemic adverse effects [38]. Advantages of this route of gene transfer include rapid onset of action, high local gene concentration, delivery directly to the target lung tissue, and long gene construct lifetime in the lung instead of quickly metabolization and excretion [39]. We believe that intra-tracheal delivery is the appropriate type of delivery to address PAH disease.

*AC mechanism of action* One of the hurdles for progress in PH treatment is the lack of comprehensive theories that explain the pathogenesis and diverse features of the disease. Pulmonary vascular cell senescence is one of the dominant theories that can explain the irreversible nature of PH and its complications. Currently cellular senescence is known as the arrest of normal cell division in response to a variety of stresses or chronic conditions [40]. In this process, cells lose their ability to preserve normal function or replicate for tissue repair, accompanied by the expression of an inflammatory senescent-associated secretory phenotype and mediators like cytokines that lead to tissue damage. We found that PH with RV dysfunction is associated with a switch to a senescent vascular phenotype, which was confirmed by demonstrating increased expression of senescence markers such as p21^Cip1^ and MMP3 in the lungs of PH-established animals.

Here we demonstrated the feasibility, efficiency, and safety of airway distribution and transduction of aerosolized Anc80L65.AC as a gene therapy to the pulmonary vessels. In this study, AC overexpression improved cardiopulmonary hemodynamics and RV parameters in rodents PH model. The response to AC enabled us to identify that this treatment can improve pulmonary remodeling decreasing medial wall hypertrophy and intimal hyperplasia. Cell blood count and chemistry values for AC group were within normal range and did not demonstrate any signs of toxicity or adverse effects on other organs. This data indicates that Anc80L65.AC gene therapy prevents the progression of PH and confirms the potential therapeutic role of AC as a novel target in PAH. Our data also extends the “proof-of-concept” strategy of aerosolized gene transfer of AC by demonstrating efficacy in a small animal model of PAH. As there are no established therapies that target the senescence process that leads to angioproliferation, apoptosis and vascular remodeling, our findings offer an innovative option to prevent PH progression.

*Study limitations* The concept that remains uncertain is which pulmonary cell type senescence is first induced and which senescent cell type contributes primarily to irreversibility in PH with RV dysfunction. In this study, we did not compare animals with different routes of gene delivery and did not analyze different viral genome copy doses of AAV viral vector and time-dependent effect. We are planning to make a single cell transcriptase analysis to explore the mechanism of lung cell senescence and cytotoxicity leading to PH phenotype.

## Conclusions

Sphingolipids metabolism pathways represent the main frontiers in the study of molecular cardiology. Gene transfer via intra-tracheal administration of AC can balance the physiological level of ceramide, provide tropism to the pulmonary tissue, ameliorate vascular remodeling, and prevent RV myocardial dysfunction. Combining Anc80L65 technology with AC gene cassette offers a robust solution for PH patients.

### Supplementary Information

**Additional file 1.** Video with comments.

**Additional file 2: Table S1.** Summary of Hemodynamic data (MRI+ RV catheterization). All continuous data were checked for normality and are presented as mean ± SD. COi, cardiac output index; HR, heart rate; LVEDVi, left ventricle end-diastolic volume index; LVESVi, left ventricle end-systolic volume index; LVEF, left ventricle ejection fraction; LV mass, left ventricle mass; mPAP, mean pulmonary arterial pressure; PVR, pulmonary vascular resistance; TAPSE, tricuspid annular plane systolic excursion; RV mass, right ventricle mass; RVEDVi, right ventricle end-diastolic volume index; LVESVi, right ventricle end-systolic volume index; RVEF, right ventricle ejection fraction. P values are listed under each group excluding baseline. The p values are obtained through a one-tail T test comparing baseline values and 4-, and 8-week time points. The p values correspond to that specific group and its baseline. **Table S2.** Summary of blood tests and blood gas analysis. All continuous data were checked for normality and are presented as mean ± SD. The following variables were assessed: red blood cells (RBC) count, hemoglobin (Hgb) concentration, packed cell volume (PCV), total white blood cells (WBC) count, percentage of neutrophils, lymphocytes, monocytes, eosinophils, and blood gases, blood urea nitrogen (BUN), aspartate aminotransferase (AST), alanine aminotransferase (ALT). The pH (measure of acidity), pCO2 (partial pressure of carbon dioxide), pO2 (partial pressure of oxygen) was carried out in blood samples as well. P values are listed under each group excluding baseline. The p values are obtained through a one-tail T test comparing baseline values to 8-week time points. The p values correspond to that specific group and its baseline.

**Additional file 3: Figure S1.** Representative western blot imaging of AC protein in lungs after Anc.80AC gene therapy in rats 8 weeks post-PH development.

**Additional file 4: Table S3.** Summary of pro-inflammatory and anti-inflammatory cytokines expression. All continuous data were checked for normality and are presented as mean ± SD. Epo, erythropoietin; G-CSF, granulocyte-colony stimulating factor; GLP-1, glucagon-like peptide-1; CM CSF, granulocyte-macrophage colony-stimulating factor; GRO KC/CXCL-1, keratinocyte chemoattractant; IFN g, interferon gamma; IL-1 α, interleukin 1 alpha; IL-1 β, interleukin 1 beta; IL-2, interleukin 2; IL-4, interleukin 4; IL-5, interleukin 5; IL-6, interleukin 6; IL-7, interleukin 7; IL-10, interleukin 10; IL-12, interleukin 12; IL-13, interleukin 13; IL-17, interleukin 17; IL-18, interleukin 18; M-CSF, macrophage colony-stimulating factor; MCP-1/CCL2, monocyte chemoattractant protein 1; MIP-3/C CCL20, macrophage inflammatory protein 3; RANTES, chemokine ligand 5, TNF-α, tumor necrosis factor alpha. P values are listed under each group excluding baseline. The p values are obtained through a one-tail T test comparing baseline values to 8-week time points. The p values correspond to that specific group and its baseline.

## Data Availability

The datasets used and/or analyzed during the current study are available from the corresponding author on reasonable request.

## Abbreviations

AAV: Adeno-associated virus
AC: Acid ceramidase
Anc80L65: Synthetic adeno-associated virus lineage clone
qPCR: Quantitative polymerase chain reaction
PAH: Pulmonary arterial hypertension
PVR: Pulmonary vascular resistance
MMP3: Matrix metalloproteinase-3 enzyme
p21^Cip1^: Cyclin-dependent kinase inhibitor 1
RV: Right ventricle
LV: Left ventricle
MRI: Magnetic resonance imaging
