# Supplementary material for: Acid ceramidase gene therapy ameliorates pulmonary arterial hypertension with right heart dysfunction
Source: Respir Res. 2023 Aug 11;24:197. doi: 10.1186/s12931-023-02487-2 (PMC10416391; doi:10.1186/s12931-023-02487-2)
Supplement: Supplementary file 2 — Additional file 2: Table S1. Summary of Hemodynamic data (MRI+ RV catheterization). All continuous data were checked for normality and are presented as mean ± SD. COi, cardiac output index; HR, heart rate; LVEDVi, left ventricle end-diastolic volume index; LVESVi, left ventricle end-systolic volume index; LVEF, left ventricle ejection fraction; LV mass, left ventricle mass; mPAP, mean pulmonary arterial pressure; PVR, pulmonary vascular resistance; TAPSE, tricuspid annular plane systolic excursion; RV mass, right ventricle mass; RVEDVi, right ventricle end-diastolic volume index; LVESVi, right ventricle end-systolic volume index; RVEF, right ventricle ejection fraction. P values are listed under each group excluding baseline. The p values are obtained through a one-tail T test comparing baseline values and 4-, and 8-week time points. The p values correspond to that specific group and its baseline. Table S2. Summary of blood tests and blood gas analysis. All continuous data were checked for normality and are presented as mean ± SD. The following variables were assessed: red blood cells (RBC) count, hemoglobin (Hgb) concentration, packed cell volume (PCV), total white blood cells (WBC) count, percentage of neutrophils, lymphocytes, monocytes, eosinophils, and blood gases, blood urea nitrogen (BUN), aspartate aminotransferase (AST), alanine aminotransferase (ALT). The pH (measure of acidity), pCO2 (partial pressure of carbon dioxide), pO2 (partial pressure of oxygen) was carried out in blood samples as well. P values are listed under each group excluding baseline. The p values are obtained through a one-tail T test comparing baseline values to 8-week time points. The p values correspond to that specific group and its baseline. [file 12931_2023_2487_MOESM2_ESM.docx]

| Table S1: Summary of blood tests and blood gas analysis at 8 weeks post-gene delivery | | | | | |
| --- | --- | --- | --- | --- | --- |
| Measure | **Sham** | **PH** | **PH.Anc80.Null** | **PH.Saline** | **PH.Anc80.AC** |
| RBC (million/μl) | 7.79±0.341 | 6.54 ±0.394  *p=0.0005* | 6.136±0.220  *p<0.0001* | 6.472±0.516  *p=0.0009* | 7.044±0.654  *p=0.0291* |
| Hgb (g/dl) | 12.46±1.00 | 10.463±0.637  *p=0.0044* | 10.35±0.945  *p=0.0045* | 10.14±0.997  *p=0.0032* | 11.54±0.403  *p=0.0466* |
| PCV (%) | 45.3 ±3.28 | 39.42±2.39  *p=0.0060* | 36.38±4.187  *p=0.0028* | 37.16±3.70  *p=0.0031* | 41.68±2.57  *p=0.0443* |
| WBC (x10^9^/L) | 7.04±0.548 | 10.77±0.81  *p<0.0001* | 13.86±1.16  *p<0.0001* | 11.23±1.29  *p<0.0001* | 8.92±1.48  *p=0.0157* |
| Neutrophils(%) | 9.03±0.79 | 12.03±0.83  *p=0.0002* | 14.62±1.01  *p<0.0001* | 12.92±1.45  *p=0.0004* | 10.52±1.53  *p=0.0442* |
| Lymphocytes (%) | 84.36±7.19 | 101.1±4.77  *p=0.0012* | 116.2±6.57  *p<0.0001* | 103.4±7.82  *p=0.0020* | 93.0±11.42  *p=ns* |
| Monocytes (%) | 2.78±0.36 | 3.42±0.711  *p=ns* | 4.52±0.585  *p=0.0002* | 3.714±1.04  *p=0.0473* | 3.2±0.538  *p=ns* |
| Eosinophils (%) | 0.262±0.051 | 0.544±0.081  *p<0.0001* | 0.742±0.075  *p<0.0001* | 0.598±0.125  *p=0.0003* | 0.39±0.092  *p=0.0120* |
| Glucose (mg/dL) | 101.2±16.3 | 114.4±10.16  *p=ns* | 101.2±12.23  *p=ns* | 109.2±10.25  *p=ns* | 96.8±8.43  *p=ns* |
| BUN (mg/dL) | 23.8±2.68 | 27.8±4.26  *p=ns* | 29.2±3.49  *p=0.0127* | 26.4±4.15  *p=ns* | 26.2±4.08  *p=ns* |
| AST (U/L) | 196±13.4 | 225.2±6.22  *p=0.0012* | 231.2±7.39  *p=0.0005* | 222.0±4.30  *p=0.0017* | 203.8±10.63  *p=ns* |
| ALT (U/L) | 84.4±11.2 | 108.8±13.49  *p=0.0073* | 101.4±9.12  *p=0.0153* | 104.2±11.9  *p=0.0136* | 89.8±7.66  *p=ns* |
| Creatinine (mg/dL) | 0.38±0.061 | 0.466±0.071  *p=0.0405* | 0.57±0.084  *p=0.0019* | 0.484±0.063  *p=0.0162* | 0.452±0.066  *p=ns* |
| Blood pH | 7.34±0.036 | 7.26±0.068  p=0.0246 | 7.24±0.032  *p=0.0007* | 7.32±0.046  *p=ns* | 7.3±0.051  *p=ns* |
| pCO2 (mm Hg) | 41.5±4.48 | 33.5±2.58  *p=0.0043* | 27.1±1.51  *p<0.0001* | 28.8±6.72  *p=0.0039* | 35.4±1.81  *p=0.0109* |
| PO2 (mm Hg) | 96.6±1.81 | 89.2±1.64  *p<0.0001* | 85.6±3.36  *p=0.0001* | 91.6±2.21  *p=0.0025* | 93.1±2.96  *p=0.0296* |
| Bicarbonate (mmol/l) | 26.1±1.64 | 21.9±2.67  *p=0.0091* | 19.6±1.40  *p<0.0001* | 22.4±1.93  *p=0.0064* | 23.4±2.39  *p=0.0394* |
| Sodium (mmol/l) | 140.2±6.94 | 127.6±6.65  *p=0.0094* | 126.6±4.77  *p=0.0034* | 129.3±5.54  *p=0.0125* | 135.7±4.15  *p=ns* |
| Potassium (mmol/l) | 3.56±0.23 | 3.24±0.16  *p=0.0179* | 3.18±0.11  *p=0.0058* | 3.24±0.16  *p=0.0193* | 3.38±0.21  *p=ns* |

|  | | |  | |  |  | |  |  | |
| --- | --- | --- | --- | --- | --- | --- | --- | --- | --- | --- |
| **Table S2.** **Summary of pro-inflammatory and anti-inflammatory cytokines** **expression** **at 8 weeks post- delivery** | | | | | | | | | | |
| **Measure**  (pg/mL) | | | **Sham** | | **PAH** | **PAH.Anc80.Null** | | **PAH.Saline** | **PAH.Anc80.AC** | |
| IL-1 β | 130.8±40.8 | | | 235.2±91.5  *p=0.0029* | | | 304.4±63.8  *p<0.0001* | 346.5±80.82  *p<0.0001* | 171.1±42.6  *p=0.0406* |  |
| TNF α | | 113.73±52.5 | | 263.7±64.0  *p<0.0001* | | | 345.5±89.3  *p<0.0001* | 383.6±64.3  *p<0.0001* | 189.7±80.3  *p=0.0186* |  |
| IFN γ | | 94.3±35.0 | | 261.0±98.8  *p<0.0001* | | | 352.8±89.3  *p<0.0001* | 334.6±70.4  *p<0.0001* | 168.3±61.6  *p=0.0040* |  |
| IL-6 | | 140.2±40.7 | | 323.1±90.5  *p<0.0001* | | | 362.5±98.3  *p<0.0001* | 282.7±81.0  *p=0.0002* | 205.4±56.8  *p=0.0090* |  |
| IL-1 α | | 137.9±82.1 | | 292.6±84.5  *p=0.0009* | | | 373.1±80.2  *p<0.0001* | 352.6±88.0  *p=0.0001* | 172.9±51.5  *p=ns* |  |
| IL-18 | | 2497±863.2 | | 3729.4±968.3  *p=0.0074* | | | 3941.2±979.6  *p=0.0029* | 4238.6±720.7  *p=0.0005* | 3091.0±1041.6  *p=ns* |  |
| MCP-1/CCL2 | | 685.1±143.2 | | 990.7±132.0  *p=0.0002* | | | 1002.2±139.9  *p=0.0002* | 1290.4±683.9  *p=0.0077* | 900.9±200.7  *p=0.0124* |  |
| GM-CSF | | 156.7±61.8 | | 264.0±94.5  *p=0.0062* | | | 298.3±92.8  *p=0.0009* | 301.3±43.9  *p<0.0001* | 240.7±54.2  *p=0.0079* |  |
| IL-17 | | 85.9±31.3 | | 106.5±65.4  *p=ns* | | | 128.1±55.0  *p=0.0311* | 158.3±60.91  *p=0.0034* | 111.9±31.1  *p=ns* |  |
| M-CSF | | 247.3±56.1 | | 302.0±61.4  *p=0.0381* | | | 289.7±31.6  *p=0.0461* | 306.0±60.6  *p=0.0348* | 241.2±82.1  p*=ns* |  |
| MIP-3/CCL20 | | 50.1±13.3 | | 87.0±30.3  *p=0.0018* | | | 113.7±54.8  *p=0.0014* | 158.1±58.1  *p<0.0001* | 97.3±31.3  *p=0.0004* |  |
| RANTES | | 260.5±84.2 | | 344.6±91.4  *p=0.0346* | | | 324.9±111.5  *p=ns* | 355.2±57.9  *p=0.0150* | 249.4±93.9  *p=ns* |  |
| IL-12 | | 94.9±46.1 | | 139.8±97.2  *p=ns* | | | 191.1±87.2  *p=0.0048* | 217.7±84.4  *p=0.0010* | 144.3±26.2  *p=0.0160* |  |
| EPO | | 524±134.8 | | 721.8±116.9  *p=0.0034* | | | 708.6±85.2  *p=0.0031* | 695.4±109.9  *p=0.0100* | 592.4±50.6  *p=ns* |  |
| G-CSF | | 20.6±5.70 | | 43.0±13.6  *p<0.0001* | | | 38.0±10.5  *p=0.0002* | 44.5±8.91  *p<0.0001* | 39.7±13.8  *p=0.0008* |  |
| GRO KC | | 162.9±79.2 | | 218.6±61.0  *p=ns* | | | 259.2±81.6  *P=0.0139* | 253.9±89.7  *p=0.0262* | 230.3±75.1  *p=ns* |  |
| GLP-1 | | 26921±899.0 | | 3488.9±990.6  *p=ns* | | | 3116±410.0  *p=ns* | 3586±855.7  *p=0.0351* | 3487±478.1  *p=ns* |  |
| IL-2 | | 363.8±88.0 | | 431.5±95.8  *p=ns* | | | 456.3±74.7  *p=0.0195* | 481.6±126.2  *p=0.0222* | 392.1±62.9  *p=ns* |  |
| IL-4 | | 62.6±19.6 | | 92.5±26.5  *p=0.0086* | | | 100.1±24.7  *p=0.0017* | 115.0±33.6  *p=0.0007* | 82.9±35.1  *p=ns* |  |
| IL-5 | | \| 220.5±60.3 \| \| --- \| \|  \| | | 297.3±84.4  *p=0.0221* | | | 372.4±95.1  *p=0.0005* | 381.4±92.5  *p=0.0004* | 249.5±79.8  *p=ns* |  |
| IL-7 | | 264.8±64.1 | | 349.6±58.5  *p=0.0070* | | | 367.7±99.7  *p=0.0100* | 337.3±83.4  *p=0.0350* | 336.5±71.9  *p=ns* |  |
| IL-10 | | 564.8±75.7 | | 697.1±94.9  *p=0.0030* | | | 751.2±93.8  *p=0.0002* | 864.0±79.9  *p<0.0001* | 583.8±95.0  *p=ns* |  |
| IL-13 | | 91.6±17.0 | | 148.1±55.2  *p=0.0039* | | | 166.8±51.0  *p=0.0003* | 216.2±83.0  p*=0.0002* | 125.0±56.2  *p=0.0482* |  |
