# Supplementary figures and images for: Acid ceramidase gene therapy ameliorates pulmonary arterial hypertension with right heart dysfunction
Source: Respir Res. 2023 Aug 11;24:197. doi: 10.1186/s12931-023-02487-2 (PMC10416391; doi:10.1186/s12931-023-02487-2)

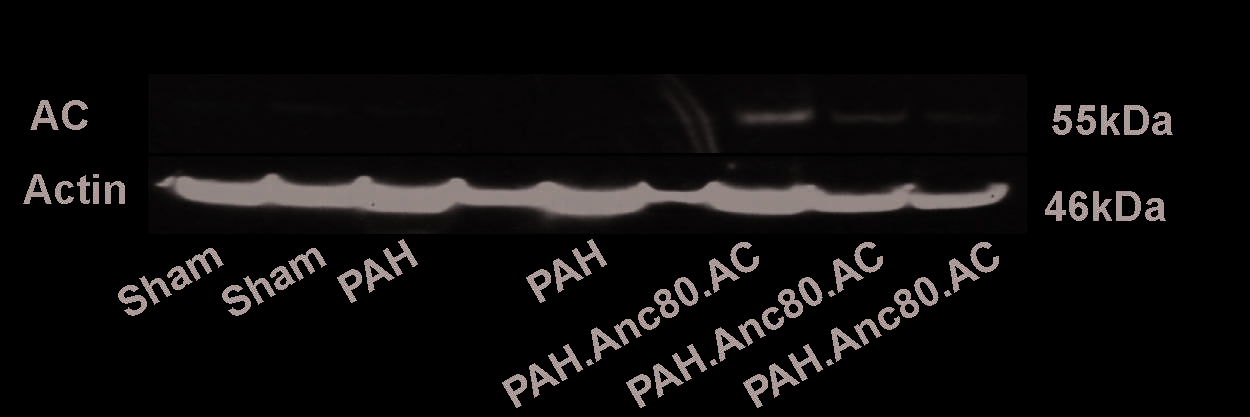

Supplement: Supplementary file 3 — Additional file 3: Figure S1. Representative western blot imaging of AC protein in lungs after Anc.80AC gene therapy in rats 8 weeks post-PH development. [file 12931_2023_2487_MOESM3_ESM.tiff]
