# Supplementary material for: Acid ceramidase gene therapy ameliorates pulmonary arterial hypertension with right heart dysfunction
Source: Respir Res. 2023 Aug 11;24:197. doi: 10.1186/s12931-023-02487-2 (PMC10416391; doi:10.1186/s12931-023-02487-2)
Supplement: Supplementary file 4 — Additional file 4: Table S3. Summary of pro-inflammatory and anti-inflammatory cytokines expression. All continuous data were checked for normality and are presented as mean ± SD. Epo, erythropoietin; G-CSF, granulocyte-colony stimulating factor; GLP-1, glucagon-like peptide-1; CM CSF, granulocyte-macrophage colony-stimulating factor; GRO KC/CXCL-1, keratinocyte chemoattractant; IFN g, interferon gamma; IL-1 α, interleukin 1 alpha; IL-1 β, interleukin 1 beta; IL-2, interleukin 2; IL-4, interleukin 4; IL-5, interleukin 5; IL-6, interleukin 6; IL-7, interleukin 7; IL-10, interleukin 10; IL-12, interleukin 12; IL-13, interleukin 13; IL-17, interleukin 17; IL-18, interleukin 18; M-CSF, macrophage colony-stimulating factor; MCP-1/CCL2, monocyte chemoattractant protein 1; MIP-3/C CCL20, macrophage inflammatory protein 3; RANTES, chemokine ligand 5, TNF-α, tumor necrosis factor alpha. P values are listed under each group excluding baseline. The p values are obtained through a one-tail T test comparing baseline values to 8-week time points. The p values correspond to that specific group and its baseline. [file 12931_2023_2487_MOESM4_ESM.docx]

|  | | |  | |  |  | |  |  | |
| --- | --- | --- | --- | --- | --- | --- | --- | --- | --- | --- |
| **Table S3.** **Summary of pro-inflammatory and anti-inflammatory cytokines** **expression** **at 8 weeks post- delivery** | | | | | | | | | | |
| **Measure**  (pg/mL) | | | **Sham** | | **PAH** | **PAH.Anc80.Null** | | **PAH.Saline** | **PAH.Anc80.AC** | |
| IL-1 β | 130.8±40.8 | | | 235.2±91.5  *p=0.0029* | | | 304.4±63.8  *p<0.0001* | 346.5±80.82  *p<0.0001* | 171.1±42.6  *p=0.0406* |  |
| TNF α | | 113.73±52.5 | | 263.7±64.0  *p<0.0001* | | | 345.5±89.3  *p<0.0001* | 383.6±64.3  *p<0.0001* | 189.7±80.3  *p=0.0186* |  |
| IFN γ | | 94.3±35.0 | | 261.0±98.8  *p<0.0001* | | | 352.8±89.3  *p<0.0001* | 334.6±70.4  *p<0.0001* | 168.3±61.6  *p=0.0040* |  |
| IL-6 | | 140.2±40.7 | | 323.1±90.5  *p<0.0001* | | | 362.5±98.3  *p<0.0001* | 282.7±81.0  *p=0.0002* | 205.4±56.8  *p=0.0090* |  |
| IL-1 α | | 137.9±82.1 | | 292.6±84.5  *p=0.0009* | | | 373.1±80.2  *p<0.0001* | 352.6±88.0  *p=0.0001* | 172.9±51.5  *p=ns* |  |
| IL-18 | | 2497±863.2 | | 3729.4±968.3  *p=0.0074* | | | 3941.2±979.6  *p=0.0029* | 4238.6±720.7  *p=0.0005* | 3091.0±1041.6  *p=ns* |  |
| MCP-1/CCL2 | | 685.1±143.2 | | 990.7±132.0  *p=0.0002* | | | 1002.2±139.9  *p=0.0002* | 1290.4±683.9  *p=0.0077* | 900.9±200.7  *p=0.0124* |  |
| GM-CSF | | 156.7±61.8 | | 264.0±94.5  *p=0.0062* | | | 298.3±92.8  *p=0.0009* | 301.3±43.9  *p<0.0001* | 240.7±54.2  *p=0.0079* |  |
| IL-17 | | 85.9±31.3 | | 106.5±65.4  *p=ns* | | | 128.1±55.0  *p=0.0311* | 158.3±60.91  *p=0.0034* | 111.9±31.1  *p=ns* |  |
| M-CSF | | 247.3±56.1 | | 302.0±61.4  *p=0.0381* | | | 289.7±31.6  *p=0.0461* | 306.0±60.6  *p=0.0348* | 241.2±82.1  p*=ns* |  |
| MIP-3/CCL20 | | 50.1±13.3 | | 87.0±30.3  *p=0.0018* | | | 113.7±54.8  *p=0.0014* | 158.1±58.1  *p<0.0001* | 97.3±31.3  *p=0.0004* |  |
| RANTES | | 260.5±84.2 | | 344.6±91.4  *p=0.0346* | | | 324.9±111.5  *p=ns* | 355.2±57.9  *p=0.0150* | 249.4±93.9  *p=ns* |  |
| IL-12 | | 94.9±46.1 | | 139.8±97.2  *p=ns* | | | 191.1±87.2  *p=0.0048* | 217.7±84.4  *p=0.0010* | 144.3±26.2  *p=0.0160* |  |
| EPO | | 524±134.8 | | 721.8±116.9  *p=0.0034* | | | 708.6±85.2  *p=0.0031* | 695.4±109.9  *p=0.0100* | 592.4±50.6  *p=ns* |  |
| G-CSF | | 20.6±5.70 | | 43.0±13.6  *p<0.0001* | | | 38.0±10.5  *p=0.0002* | 44.5±8.91  *p<0.0001* | 39.7±13.8  *p=0.0008* |  |
| GRO KC | | 162.9±79.2 | | 218.6±61.0  *p=ns* | | | 259.2±81.6  *P=0.0139* | 253.9±89.7  *p=0.0262* | 230.3±75.1  *p=ns* |  |
| GLP-1 | | 26921±899.0 | | 3488.9±990.6  *p=ns* | | | 3116±410.0  *p=ns* | 3586±855.7  *p=0.0351* | 3487±478.1  *p=ns* |  |
| IL-2 | | 363.8±88.0 | | 431.5±95.8  *p=ns* | | | 456.3±74.7  *p=0.0195* | 481.6±126.2  *p=0.0222* | 392.1±62.9  *p=ns* |  |
| IL-4 | | 62.6±19.6 | | 92.5±26.5  *p=0.0086* | | | 100.1±24.7  *p=0.0017* | 115.0±33.6  *p=0.0007* | 82.9±35.1  *p=ns* |  |
| IL-5 | | \| 220.5±60.3 \| \| --- \| \|  \| | | 297.3±84.4  *p=0.0221* | | | 372.4±95.1  *p=0.0005* | 381.4±92.5  *p=0.0004* | 249.5±79.8  *p=ns* |  |
| IL-7 | | 264.8±64.1 | | 349.6±58.5  *p=0.0070* | | | 367.7±99.7  *p=0.0100* | 337.3±83.4  *p=0.0350* | 336.5±71.9  *p=ns* |  |
| IL-10 | | 564.8±75.7 | | 697.1±94.9  *p=0.0030* | | | 751.2±93.8  *p=0.0002* | 864.0±79.9  *p<0.0001* | 583.8±95.0  *p=ns* |  |
| IL-13 | | 91.6±17.0 | | 148.1±55.2  *p=0.0039* | | | 166.8±51.0  *p=0.0003* | 216.2±83.0  p*=0.0002* | 125.0±56.2  *p=0.0482* |  |
